# Facile Fabrication of Highly Active CeO_2_@ZnO Nanoheterojunction Photocatalysts

**DOI:** 10.3390/nano13081371

**Published:** 2023-04-14

**Authors:** Xiaoqian Ai, Shun Yan, Chao Lin, Kehong Lu, Yujie Chen, Ligang Ma

**Affiliations:** 1 School of Physics and Information Engineering, Jiangsu Province Engineering Research Center of Basic Education Big Data Application, Jiangsu Second Normal University, Nanjing 210013, China; xqai186@jssnu.edu.cn (X.A.);; 2 School of Electronic Engineering, Nanjing Xiaozhuang University, Nanjing 211171, China

**Keywords:** zinc oxide, cerium oxide, nanocomposites, photocatalysis, heterojunction

## Abstract

Photocatalyst performance is often limited by the poor separation and rapid recombination of photoinduced charge carriers. A nanoheterojunction structure can facilitate the separation of charge carrier, increase their lifetime, and induce photocatalytic activity. In this study, CeO_2_@ZnO nanocomposites were produced by pyrolyzing Ce@Zn metal–organic frameworks prepared from cerium and zinc nitrate precursors. The effects of the Zn:Ce ratio on the microstructure, morphology, and optical properties of the nanocomposites were studied. In addition, the photocatalytic activity of the nanocomposites under light irradiation was assessed using rhodamine B as a model pollutant, and a mechanism for photodegradation was proposed. With the increase in the Zn:Ce ratio, the particle size decreased, and surface area increased. Furthermore, transmission electron microscopy and X-ray photoelectron spectroscopy analyses revealed the formation of a heterojunction interface, which enhanced photocarrier separation. The prepared photocatalysts show a higher photocatalytic activity than CeO_2_@ZnO nanocomposites previously reported in the literature. The proposed synthetic method is simple and may produce highly active photocatalysts for environmental remediation.

## 1. Introduction

The continued use of fossil fuels has resulted in a global energy crisis, environmental pollution, and climate change [1,2], thus, more sustainable energy resources are essential to combat these issues [3]. Photocatalytic technologies [4,5] for exploiting light energy have drawn particular attention [6,7,8]. Such catalysts can be used to produce hydrogen from water for energy storage and convert the excess carbon dioxide in the atmosphere to valuable chemical feedstocks and fuels, such as methane and methanol [9,10,11,12,13,14]. Furthermore, photocatalytic materials can be used to degrade organic pollutants in contaminated water [15,16,17]. In particular, compared with traditional methods for water treatment, photocatalysis requires less energy and can achieve a complete degradation of pollutants. Therefore, photocatalytic systems are highly promising for clean energy production and environmental remediation. To achieve high photocatalytic activity, photoinduced charge carriers (i.e., electrons and holes) must be effectively generated and separated in the photocatalysts.

Several photocatalytic mechanisms for the degradation of organic pollutants such as dyes have been reported. Briefly, upon irradiation with ultraviolet light, electrons in the valence band (VB) are excited to the conduction band (CB); thus, holes are created in the VB. The electrons in the CB react with adsorbed oxygen to form superoxide radicals (•O_2_^−^), whereas the holes in the VB react with water to form hydroxyl radicals (•OH), and these two radicals react with organic pollutants and degrade them. To date, many photocatalysts have been reported, including CdS [18,19], ZnO [20], CeO_2_ [21], TiO_2_ [22], WO_3_ [23], and graphitic carbon nitride (g-C_3_N_4_) [24]. However, the charge carriers generated by these single-phase catalysts can easily recombine, resulting in short carrier lifetimes and low catalytic efficiencies. To address this problem, multiphase catalysts, such as nanocomposites, i.e., Fe_2_O_3_/Cu_2_O [25], ZnO/TiO_2_ [26], GQDs/NiSe-NiO [27], g-C_3_N_4_/Ni-ZnO [28], and MoS_2_/TiO_2_ nanocomposites [29], have been prepared. These nanocomposites can increase the lifetimes of the charge carriers by restricting the generated electrons and holes in different phases and reducing their recombination.

Considering the matched band gaps of CeO_2_ and ZnO, we have reported on a CeO_2_@ZnO nanocomposite as an efficient photocatalyst [30]. In our previous study, a Ce@Zn-bimetallic metal–organic framework (Ce@Zn-MOF) precursor was prepared with a Zn:Ce atomic ratio of 1; subsequently, the Ce@Zn-MOF precursor was subjected to thermal decomposition to obtain photocatalytic CeO_2_@ZnO nanocomposites. The optimal pyrolysis temperature was identified as 450 °C based on the structure, morphology, and photocatalytic degradation performance of the nanocomposites. However, the effects of the Zn:Ce ratio have not been studied.

Therefore, in this study, we fabricated Ce/Zn-MOF precursors with various Zn:Ce atomic ratios (0:10, 2:8, 4.5:5.5, 6.7:3.3, 8:2, and 10:0). CeO_2_@ZnO nanocomposites were then obtained via thermal decomposition at the previously identified optimal temperature (450 °C). The structure, morphology, and optical properties of CeO_2_, ZnO, and the CeO_2_@ZnO nanocomposites were investigated via X-ray diffractometry (XRD), scanning electron microscopy (SEM), transmission electron microscopy (TEM), and UV-vis absorption spectroscopy. In addition, the prepared nanocomposites were employed for photocatalytic water remediation using rhodamine B (RhB) as a model organic pollutant. Finally, the photocatalytic degradation mechanism was determined.

## 2. Experimental Method

### 2.1. Precursor and Photocatalyst Synthesis

Ce-MOF, Zn-MOF, and Ce/Zn-MOF were prepared according to our previously reported method. Briefly, Ce(NO_3_)_2_·6H_2_O (30 mmol) and 2-methylimidazole (63 mmol) were dissolved in methanol (500 mL), and the mixture was stirred, precipitated, and centrifuged to obtain Ce-MOF. To prepare the bimetallic Ce/Zn-MOF precursors Zn(NO_3_)_2_·6H_2_O and Ce(NO_3_)_2_·6H_2_O in atomic ratios of 0:10, 2:8, 4.5:5.5, 6.7:3.3, 8:2, and 10:0 were added to methanol. The samples with Zn:Ce ratios of 0:10 and 10:0 yielded Ce-MOF and Zn-MOF, respectively. Ce-MOF, Zn-MOF, and Ce/Zn-MOF were obtained via sequential precipitation, washing, centrifugation, and drying. Finally, the Ce-MOF, Zn-MOF, and Ce/Zn-MOF precursors were pyrolyzed at 450 °C in a tubular sintering furnace for 3 h to produce the CeO_2_, ZnO, and the CeO_2_@ZnO nanocomposites [30]. The annealed samples are denoted as CeO_2_@ZnO-*x*, where *x* is the ratio of Zn to Ce; for example, the sample with a ratio of 2:8 is denoted as CeO_2_@ZnO-0.2.

### 2.2. Characterization

The effect of different ratios of ZnO and CeO_2_ on the lattice structure of the CeO_2_@ZnO nanocomposites was determined using XRD (Dmax-rB, Rigaku; Tokyo, Japan, Cu-*K*_α_*λ* = 1.5418 Å) with a tube voltage and current of 40 kV and 80 mA, respectively. The changes in the morphology, microstructure, and elemental distribution of the CeO_2_@ZnO-*x* nanocomposites were observed using field emission SEM (ZEISS Gemini 500) and TEM (FEITecnai G2 F30). For TEM analysis, the CeO_2_@ZnO-*x* nanocomposites were ultrasonically dispersed in ethanol for 10 min and then dropped onto a Cu grid, and TEM observation was carried out at an acceleration voltage of 200 kV. The electronic structures and valence states of the elements were characterized using X-ray photoelectron spectroscopy (XPS). The optical band gaps of the nanocomposites were determined using UV-vis spectroscopy (UH4150, Hitachi). The spectrometer was equipped with an integrating sphere.

The photocatalytic activity of the prepared CeO_2_@ZnO nanocomposites was evaluated by measuring the degradation of RhB as a model organic pollutant using a multi-channel photochemical reaction system (PCX-50C). The light source was ultraviolet light at 365 nm with a real power density of 320 mWcm^−2^. For the degradation tests, the nanocomposites (50 mg) were ultrasonically dispersed in an aqueous RhB solution (50 mL, 10 mg/L) for 10 min. The suspension was then placed in the dark for 60 min with continuous magnetic agitation until it reached dynamic adsorption–desorption equilibrium. Before irradiation, an aliquot (3 mL) of the degradation solution was extracted and centrifuged to determine the degree of degradation. Subsequently, during light irradiation, aliquots (3 mL) were collected every 5 min and analyzed. Note that no precious metal catalyst was added during the degradation process. In addition, electron paramagnetic resonance (EPR, Bruker EMXplus) spectroscopy was used to identify the free radicals produced upon light irradiation to investigate the degradation mechanism. For these measurements, the nanomaterials were added to a 5,5-dimethyl-1-pyridine-N-oxide (DMPO) solution and mixed with deionized water or CH_3_OH to detect the concentrations of hydroxyl(•OH) and superoxide (•O_2_^−^) radicals, respectively. The transient photocurrent and electrochemical impedance spectroscopy (EIS) measurements were conducted on an electrochemical workstation (CHI660E) with three electrodes. A Pt wire and Ag/AgCl were used as the counter and reference electrodes, respectively. For the photocurrent measurement, indium tin oxide glass coated with the photocatalyst was used as the working electrode, whereas a glass-carbon electrode coated with the photocatalyst was the working electrode for the EIS measurement. A Na_2_SO_4_ solution (0.5 M) was used as the electrolyte. Photoluminescence (PL) spectra were recorded on a spectrofluorometer (Hitachi F7000) equipped with a 250 nm excitation source.

## 3. Results and Discussion

**Figure 1:**
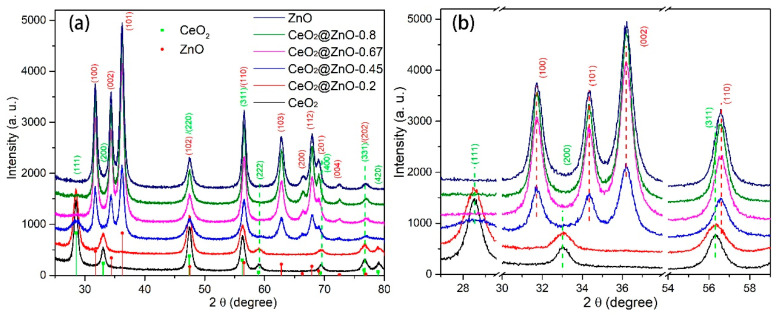
(**a**) XRD patterns for the CeO_2_, ZnO, and CeO_2_@ZnO nanocomposites (predicted peak positions for cubic CeO_2_ and hexagonal ZnO are shown on the *x*-axis) and (**b**) an enlarged figure showing the most intense diffraction peaks in (**a**).

Figure 1a shows the XRD patterns of the nanomaterials formed by the pyrolysis of the MOF precursors at 450 °C. The diffraction peaks of the nanomaterial formed by pyrolyzing Ce-MOF are indexed to cubic CeO_2_ (JCPDS Card No. 81-0792) [31]. When the Zn:Ce ratio increases to 0.2, the intensity of the CeO_2_ diffraction peaks decreases slightly (Figure 1a, CeO_2_@ZnO-0.2). With a further increase in the Zn:Ce ratio, these CeO_2_ peaks decrease in intensity, and new diffraction peaks corresponding to hexagonal wurtzite ZnO (JCPDS Card No. 36-1451) appear (Figure 1a, CeO_2_@ZnO-0.45) [32], suggesting the formation of a CeO_2_@ZnO nanocomposite. When the Zn:Ce ratios are 0.67 and 0.8 (Figure 1a, CeO@ZnO-0.67 and CeO@ZnO-0.8, respectively), the peaks corresponding to CeO_2_ disappear, and those corresponding to ZnO increase in intensity. XPS analysis revealed that some CeO_2_ was present in these two samples, and the lack of diffraction peaks is likely a result of the low quantity of CeO_2_, which resulted in these peaks being X-ray invisible or obscured by those of ZnO. Finally, the pyrolysis product of Zn-MOF displays the characteristic diffraction peaks of ZnO. The phase evolution in the nanocomposites with respect to the Zn:Ce ratio is more distinct from 2ϴ = 29° to 59° (Figure 1b), wherein the peaks corresponding to CeO_2_ gradually disappear, whereas those related to ZnO gradually intensify. Notably, the positions of the peaks corresponding to CeO_2_ and ZnO do not change with the Zn:Ce ratio, indicating that Zn did not enter the CeO_2_ lattice and vice versa.

**Figure 2:**
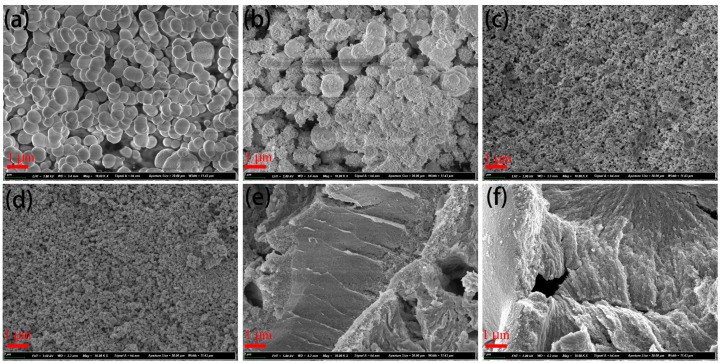
SEM images of (**a**) CeO_2_, (**b**) CeO_2_@ZnO-0.2, (**c**) CeO_2_@ZnO-0.45, (**d**) CeO_2_@ZnO-0.67, (**e**) CeO_2_@ZnO-0.8, and (**f**) ZnO.

Figure 2 shows the morphologies of the CeO_2_, ZnO, and CeO_2_@ZnO nanocomposites. As shown in Figure 2a, CeO_2_ has a smooth nanosphere morphology and uniform size distribution (approximate diameter ~800 nm). With an increase in the Zn:Ce ratio, additional nanoparticles merge on the nanospheres (Figure 2b, CeO_2_@ZnO-0.2), and the surfaces of the nanospheres become rough. For CeO_2_@ZnO-0.45 and CeO_2_@ZnO-0.67, the large nanospheres disappear, and only small nanoparticles are observed in the nanocomposites. With a further increase in the Zn:Ce ratio, the small nanoparticles aggregate, as shown in Figure 2e,f. Thus, adding Zn converts the large spheres into small nanoparticles, but excess Zn results in the aggregation of the small nanoparticles, which should decrease the specific surface area of the nanocomposites.

**Figure 3:**
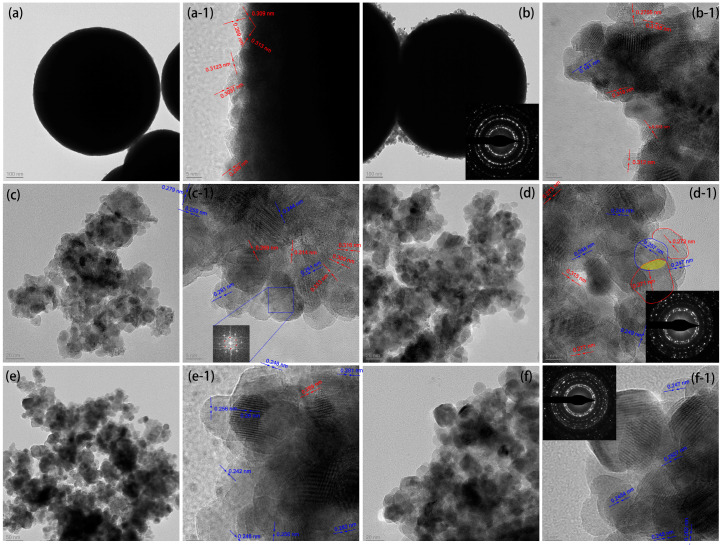
TEM images of (**a**) CeO_2_, (**b**) CeO_2_@ZnO-0.2, (**c**) CeO_2_@ZnO-0.45, (**d**) CeO_2_@ZnO-0.67, (**e**) CeO_2_@ZnO-0.8, and (**f**) ZnO. The suffix “-1” indicates the high-resolution TEM images, and the insets show the corresponding selected area electron diffraction patterns. The lattice fringes in red and blue correspond to CeO_2_ and ZnO, respectively.

The microstructures of the CeO_2_, ZnO, and CeO_2_@ZnO nanocomposites were observed using TEM. The nanospheres in Figure 3a are approximately 758 nm in diameter. In addition, the high-resolution TEM (HRTEM) images reveal that the lattice fringes at the edges of the nanospheres have interplanar distances of 0.313 and 0.269 nm, which correspond to the (111) and (200) crystal planes of cubic CeO_2_, respectively. Consistent with the SEM results, when the Zn:Ce ratio is 0.2, some nanoparticles are attached to the edges of the CeO_2_ nanospheres, while the size of the CeO_2_ nanospheres does not change significantly. These nanoparticles show lattice fringes corresponding to both cubic CeO_2_ and hexagonal wurtzite ZnO; in particular, the 0.141 nm interplanar distance corresponds to the (200) plane of ZnO. With a further increase in the Zn:Ce ratio, the number of hexagonal wurtzite ZnO nanoparticles gradually increases, whereas the number of CeO_2_ spheres in the cubic phase decreases. The diameter of the observed nanoparticles is approximately 10 nm. In addition, two sets of diffraction rings are observed in the selected area electron diffraction (SAED) pattern (Figure 3d-1). Importantly, an obvious boundary is observed between the ZnO and CeO_2_ phases, indicating the formation of a ZnO@CeO_2_ nanoheterojunction (Figure 3d-1). For the ZnO formed by the pyrolysis of the Zn-MOF precursor (Figure 3f-1), the size of the nanoparticles increases to approximately 25 nm, mainly because of the aggregation and growth of the nanoparticles at high temperatures. Its lattice fringes are detected at 0.247 and 0.2827 nm, corresponding to the (101) and (100) crystal phases of hexagonal wurtzite ZnO, respectively.

**Figure 4:**
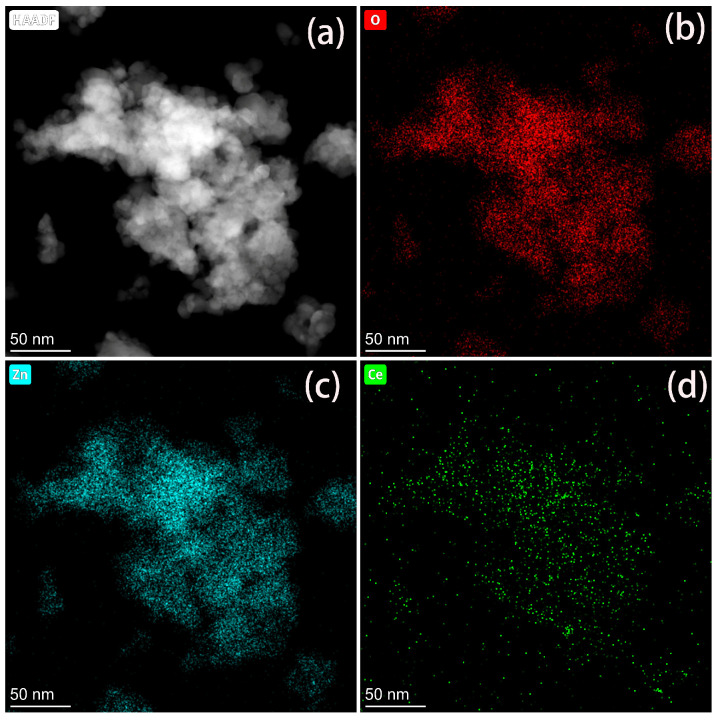
(**a**) Scanning tunneling electron microscopy high-angle annular dark field image of CeO_2_@ZnO-0.67 and the corresponding (**b**) O, (**c**) Zn, and (**d**) Ce elemental maps.

The elemental distribution in CeO_2_@ZnO-0.67 was also characterized via EDS mapping. As shown in Figure 4, Zn, Ce, and O are distributed uniformly in the nanocomposite. However, the distribution of Ce is sparser than those of Zn and O, which is consistent with the high Zn content in CeO_2_@ZnO-0.67.

**Figure 5:**
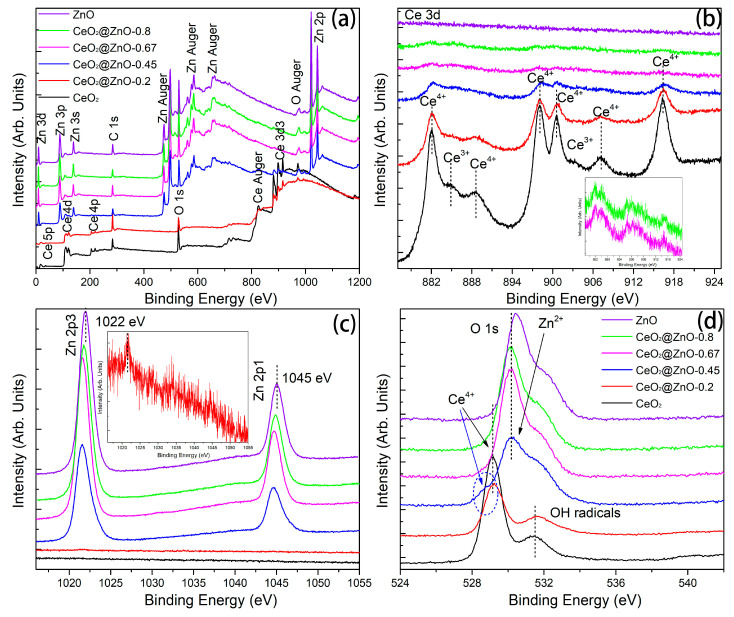
XPS spectra of the CeO_2_, ZnO, and CeO_2_@ZnO nanocomposites: (**a**) survey spectra and high-resolution (**b**) Ce 3d, (**c**) Zn 2p, and (**d**) O 1s spectra.

XPS measurements were conducted to investigate the elemental composition and surface chemical valence states. The C 1s peak is related to the adventitious carbon introduced during pyrolysis. Therefore, the spectra were calibrated based on the C=C peak at a binding energy of 285.0 eV. As expected, the XPS survey spectra of the CeO_2_@ZnO nanocomposites contain peaks corresponding to Ce, Zn, and O. In the Ce 3d high-resolution XPS spectra of CeO_2_ (black curve in Figure 5b), the Ce^3+^ and Ce^4+^ peaks are detected [33,34,35], indicating the presence of CeO_2_ and Ce_2_O_3_. The Ce_2_O_3_ phase was not detected via XRD, possibly because of its amorphous nature. In contrast, in the XPS spectra of the CeO_2_@ZnO nanocomposites, the Ce^3+^ peaks are weakened until they disappear, indicating that Ce is present exclusively in the CeO_2_ phase. The split peaks in the high-resolution Zn 2p spectra correspond to Zn 2p_3/2_ and 2p_1/2_, respectively, indicating that Zn is present as Zn^2+^ [36]. Interestingly, the Zn peaks in the CeO_2_@ZnO nanocomposites are at lower binding energies than those in ZnO, suggesting the formation of an interface between CeO_2_ and ZnO. In the high-resolution O 1s spectra (Figure 5d), three peaks at 529.1, 530.2, and 531.6 eV can be assigned to the oxygen bonded to Ce, Zn, and surface hydroxyl radicals [33,37,38], respectively. Overall, the XPS and HRTEM results indicate that a heterojunction interface between ZnO and CeO_2_ nanoparticles is formed via pyrolysis. The interface is crucial for photocatalytic applications because photoelectrons can migrate across the interface and be effectively separated from the photogenerated holes.

**Figure 6:**
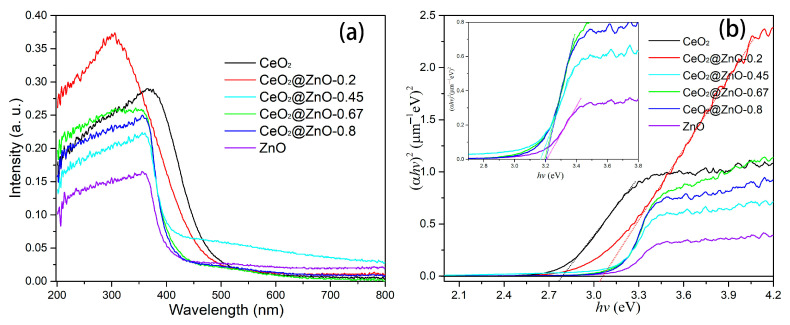
(**a**) UV-visible absorption spectra and (**b**) plots of (*αhν*)^2^ vs. *hν* for the CeO_2_, ZnO, and CeO_2_@ZnO nanocomposites.

The optical properties of the CeO_2_, ZnO, and CeO_2_@ZnO nanocomposites were investigated using UV-vis absorption spectroscopy, and the results are shown in Figure 6a. With an increase in the Zn:Ce ratio, the optical absorption edge shows a progressive blue shift. The optical band gaps of the CeO_2_, ZnO, and CeO_2_@ZnO nanocomposites were obtained using the Tauc formula [39]: *αhν* = *A*(*hν* − *E*_g_)^2^, where *α*, *h*, *ν*, *A*, and *E*_g_ are the absorption coefficient, Planck’s constant, the frequency of the incident light, a constant, and the optical band gap, respectively. The fitting curves for (*αhν*)^2^ vs. *hν* are shown in Figure 6b. The linear part of the curve is extrapolated, and the x intersection is the optical band gap. Thus, the optical band gaps of the CeO_2_, CeO_2_@ZnO-0.2, CeO_2_@ZnO-0.45, CeO_2_@ZnO-0.67, CeO_2_@ZnO-0.8, and ZnO nanomaterials are 2.789, 3.06, 3.164, 3.195, 3.20, and 3.214 eV, respectively, showing an increasing trend with an increase in the Zn:Ce ratio. As the Zn:Ce ratio is increased, a transformation from CeO_2_ to CeO_2_@ZnO to ZnO occurs, and the size of the composite decreases from 800 nm to 10 nm. The quantum effects arising from particle size limitations cause a blue-shift in the absorption edge and an increase in the band gap. Crucially, the band gap determines the range of light that can be absorbed and, thus, used during photocatalytic degradation.

**Figure 7:**
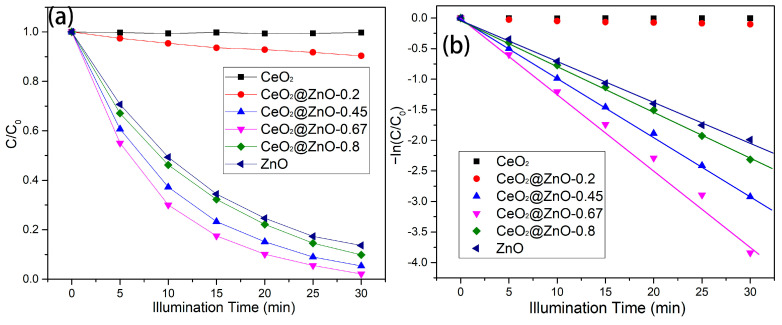
(**a**) Degradation efficiencies and (**b**) kinetic plots for the degradation of RhB over the CeO_2_, ZnO, and CeO_2_@ZnO nanocomposites during with respect to irradiation time.

The catalytic activity of the photocatalyst was evaluated by analyzing the degree of photodegradation of RhB, as calculated using Equation (1) [19]:

Photodegradation efficiency (%) = (1 − C/C_0_) × 100%, (1)

where C_0_ and C represent the UV-vis light absorption coefficients of RhB at adsorption equilibrium in the dark and upon light irradiation, respectively. Figure 7a shows the photodegradation efficiency for RhB with respect to irradiation time. The photocatalytic efficiency of CeO_2_ for RhB is very low. The smooth and large nanospheres of CeO_2_ have a low specific surface area, leading to a small contact area between CeO_2_ and RhB. The photodegradation efficiency of CeO_2_@ZnO-0.2 slightly increases, which can be attributed to the nanoparticles attached to the CeO_2_ nanospheres. With a further increase in the Zn:Ce ratio, the CeO_2_@ZnO-0.67 nanocomposite exhibits the best photocatalytic efficiency (approximately ~97% RhB degradation after 30 min of irradiation). However, the photocatalytic efficiencies of CeO_2_@ZnO-0.8 and ZnO are lower than that of the CeO_2_@ZnO-0.67.

The experimental data were fitted using the pseudo-first-order kinetic model shown in Equation (2) [30,34].

(2) $$In(\frac{C}{C_{0}})=-kt$$

Here, *k* (min^−1^) is the kinetic degradation rate constant, and *t* (min) is the reaction time. As shown in Figure 7b, the plots of −In(C/C_0_) vs. *t*. approximately follow a linear relationship, indicating that this model can be used to analyze the photodegradation rate. The degradation rates over CeO_2_ and CeO_2_@ZnO-0.2 are very low and not reported here. The kinetic degradation rate constant (*k*) values for the photodegradation of RhB over CeO_2_@ZnO-0.45, CeO_2_@ZnO-0.67, CeO_2_@ZnO-0.8, and ZnO were calculated as 0.0955, 0.124, 0.0749, and 0.0669, respectively. The CeO_2_@ZnO-0.67 nanocomposite exhibits the best photodegradation rate constant (0.124), which is superior to the highest photodegradation rate constant observed in our previous study on CeO_2_@ZnO photocatalysts (0.1096). Therefore, the optimal Zn/Ce atomic ratio was 0.67 at the optimal pyrolysis temperature (450 °C). We have summarized recent reports on the photodegradation performance of CeO_2_@ZnO, which are listed in Table 1. As can be seen from Table 1, CeO_2_@ZnO-0.67, the nanocomposites prepared by the binary MOF pyrolysis method in this paper, show better performance.

**Figure 8:**
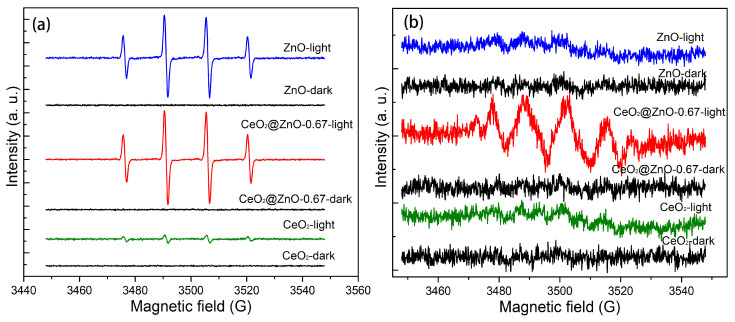
EPR spectra of the CeO_2_, ZnO, and CeO_2_@ZnO nanocomposites in the dark and under light irradiation: (**a**) DMPO-OH and (**b**) DMPO-O_2_^−^.

The production of free radicals was investigated under dark and light conditions using EPR spectroscopy. The peak intensities in the EPR spectra reflect the concentrations of free radicals. As shown in Figure 8, no free radicals are detected in the dark, whereas two types of free radicals are formed upon light irradiation. Furthermore, the concentration of free radicals produced by the CeO_2_@ZnO-0.67 nanocomposite is the highest under light irradiation, confirming that these free radicals are responsible for enhancing the photodegradation efficiency.

**Figure 9:**
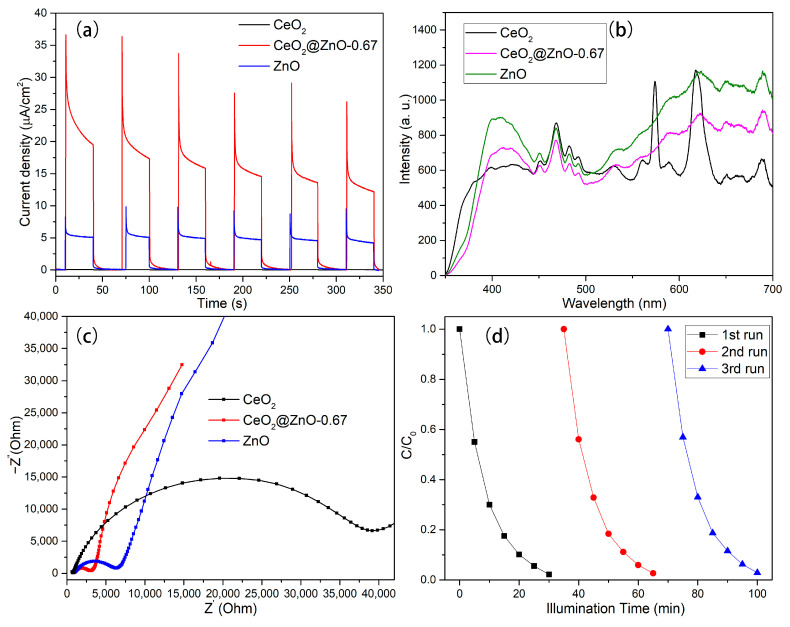
(**a**) Transient photocurrent curves, (**b**) PL spectra, (**c**) and EIS Nyquist plots for CeO, CeO_2_@ZnO-0.67, and ZnO, respectively. (**d**) Cycling experiments of the photodegradation of RhB over CeO_2_@ZnO-0.67.

To investigate the separation ability of the photogenerated electrons and holes, the transient photocurrent characteristics of the CeO, ZnO, and CeO_2_@ZnO-0.67 nanocomposites were measured. As shown in Figure 9a, the CeO_2_@ZnO-0.67 nanocomposite produces a stronger photocurrent than CeO_2_ and ZnO, indicating that has the highest number of photogenerated charge carriers under illumination. In addition, the PL spectra of the CeO_2_, ZnO, and CeO_2_@ZnO-0.67 were measured. As shown in Figure 9b, the intensity of the luminescent peak for CeO_2_@ZnO-0.67 is significantly lower than that of CeO_2_ and ZnO, which indicates that the electron–hole pairs generated by CeO_2_@ZnO-0.67 have a low recombination rate. The efficiency of the direct electron transfer and separation of photogenerated electrons was evaluated using EIS (Figure 9c). In the EIS spectra, the arc radius determines the resistance of the interface layer, which affects the separation of electrons. A small arc radius means that electrons can be transported quickly. Among the three photocatalysts, CeO_2_@ZnO-0.67 exhibits the smallest arc radius, indicating its excellent charge transfer ability. Cycling experiments were performed to evaluate the stability and recyclability of CeO_2_@ZnO-0.67. Figure 9d shows that the high photodegradation efficiency of CeO_2_@ZnO-0.67 is maintained after three cycles.

**Figure 10:**
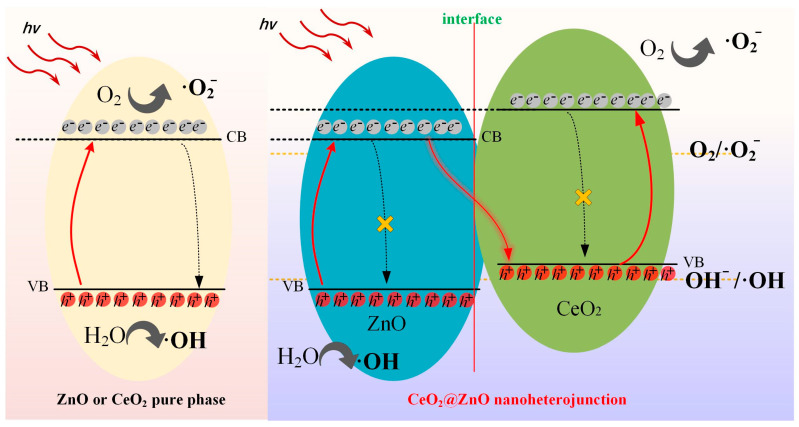
Separation mechanisms of photogenerated electron–hole pairs of the pure phase materials and the CeO_2_@ZnO nanoheterojunction.

Based on the previously described analysis, the separation mechanism of the photogenerated electron–hole pairs is shown in Figure 10. Generally, the photoexcited electrons easily recombine with the holes in the VB. In CeO_2_ or ZnO pure phase materials, the recombination of excited electrons in CB and holes in VB is dominant, which considerably reduces the efficiency of photodegradation. Therefore, the inhibition of charge carrier recombination is crucial for enhancing photocatalytic efficiency. Unlike CeO_2_ and ZnO, the CeO_2_@ZnO nanocomposite contains a heterojunction interface, which prevents the recombination of photogenerated charge carriers and ensures the production of free radicals for Z-scheme catalytic photodegradation. In detail, the excited electrons in ZnO preferentially recombine with the holes in CeO_2_, which enables the electrons in the CB of CeO_2_ and the holes in the VB of ZnO to interact fully with oxygen and water to generate free radicals for dye decomposition.

## 4. Conclusions

In this study, CeO_2_@ZnO nanocomposites with various Zn:Ce ratios were prepared via the pyrolysis of Ce/Zn-MOFs precursors. As the Zn:Ce ratio increases from zero to one, pure CeO_2_, CeO_2_@ZnO nanocomposites, and pure ZnO are obtained. Pure CeO_2_ exists as nanospheres with diameters of approximately 800 nm. With an increase in the Zn:Ce ratio, the CeO_2_@ZnO nanocomposites gradually transform from nanospheres to nanoparticles of approximately 10 nm diameter, increasing the specific surface area. In addition, a heterojunction is formed, as evidenced by TEM and XPS analysis. The optical band gaps of the nanocomposites widen with an increase in the Zn:Ce ratio owing to the heterojunction interface and quantum size effects. Among the produced photocatalysts, the CeO_2_@ZnO nanocomposite with a Zn:Ce ratio of 0.67 exhibits the best photocatalytic efficiency, which is higher than that of a CeO_2_@ZnO nanocomposite with a Zn:Ce ratio of 1. In addition, this work can be extended to the preparation of other metal oxide nanocomposites, and excellent photocatalytic performance can be obtained.

## Figures and Tables

**Table 1:** Summary of different synthetic methods of CeO_2_, ZnO, and CeO_2_@ZnO nanomaterials with regard to photodegradation.

Photocatalyst	Synthetic Method	Morphology	Light Source	Catalyst Amount	Degraded Object	Illumination Time	Photodegradation Efficiency	Reference
CeO_2_@ZnO	Hydrothermal approach	Ordered mesoporous	380 nm < λ <780 nm	50 mg	MB	150 min	97.4%	[40]
CeO_2_@ZnO	Electrospinning technique	Nanofibers	365 nm	10 mg	RhB	180 min	98%	[41]
CeO_2_@ZnO	Sol–gel method	Nanocomposites	>420 nm	50 mg	RhB	250 min	50%	[42]
CeO_2_/ZnO@Au	Hydrothermal method	Hierarchical heterojunction	Xe lamp	10 mg	RhB	20 min	99%	[43]
CuO/CeO_2_/ZnO	Two-step sol–gel method	Nanoparticles	UV light	50 mg	RhB	30 min	98%	[44]
CeO_2_/ZnO	In situ precipitation method	Nanocomposites	UV light	50 mg	RhB	80 mn	42%	[45]
CeO_2_/ZnO	Pyrolyzing Ce@Zn metal–organic frameworks	Nanoheterojunction	UV light	50 mg	RhB	30 min	97%	This work

## Data Availability

Not applicable.
